# SorghumWeedDataset_Classification and SorghumWeedDataset_Segmentation datasets for classification, detection, and segmentation in deep learning

**DOI:** 10.1016/j.dib.2023.109935

**Published:** 2023-12-09

**Authors:** Michael J. Justina, M. Thenmozhi

**Affiliations:** a Department of Computer Science and Engineering, School of Computing, SRM Institute of Science and Technology, Kattankulathur Campus, Chennai 603203, India; b Department of Networking and Communications, School of Computing, SRM Institute of Science and Technology, Kattankulathur Campus, Chennai 603203, India

**Keywords:** Uniform and random crop-spacing, Crop-weed identification, Crop-weed dataset, Sorghum_dataset, Research_data, Precision agriculture, Autonomous weeding, Machine learning and deep learning

## Abstract

The intuition behind this data acquisition is to encourage research for addressing the problem of weeds in agriculture through computer vision applications. Data is acquired in the form of images from uniform and random crop-spacing fields. In other words, we have taken a step forward to identify weeds from fields that follow any method of sowing, which ultimately leads to the transformation of traditional agriculture into precision agriculture. Sorghum crop and its associated weeds are chosen as the research objects during this process. These acquired data are used in framing two datasets. The first dataset termed ‘SorghumWeedDataset_Classification’ is a crop-weed classification dataset created with 4312 data samples for addressing crop-weed classification problems. The second dataset termed ‘SorghumWeedDataset_Segmentation’ is a crop-weed segmentation dataset that contains 5555 manually pixel-wise annotated data segments from 252 data samples for addressing crop-weed localization, detection, and segmentation problems. All data samples are acquired in April and May 2023 from Sri Ramaswamy Memorial (SRM) Care Farm, Chengalpattu district, Tamil Nadu, India. Manually annotated data samples and data segments are verified by agronomists. The datasets are made publicly available to the research community to solve the crop-weed problems using state-of-the-art image processing, machine learning, and deep learning algorithms. To the best of our knowledge, these are the first open-access crop-weed research datasets from Indian fields for classification and segmentation to deal with weed issues in uniform and random crop-spacing fields. However, other available datasets (from Indian fields) are either non-research datasets or available on subscription/request.

## Graphical abstract

**Figure:**
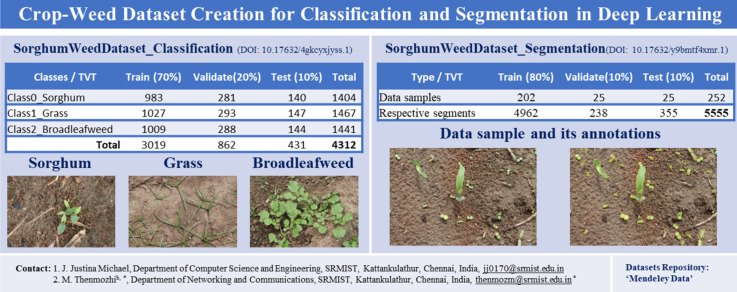
Sorghum-Weed Classification and Segmentation datasets.

Specifications Table

**Table:** 

Subject	Artificial Intelligence, Computer Vision and Pattern Recognition, Data Science, Agronomy and Crop Science, Agriculture Engineering, Precision Agriculture
Specific subject area	Crop-weed classification, identification, detection, localization, and pixel-wise segmentation using state-of-the-art deep learning/machine learning algorithms. Other similar terms: Crop-weed differentiation, Weed species identification, and segmentation from background
Data format	Raw: .jpg format (Joint Photographic Experts Group) with RGB (Red, Green, and Blue) colors
Type of data	Image
Data collection	The research objects focused are the early stages of sorghum, grasses, and broadleaved weeds. ‘SorghumWeedDataset_Classification’ contains 4312 data samples for classification and ‘SorghumWeedDataset_Segmentation’ contains 5555 manually pixel-wise annotated data segments from 252 data samples for segmentation. The instrument used is the Canon EOS 80D – a Digital Single Lens Reflex (DSLR) camera with a sensor type of 22.3 mm × 14.9 mm CMOS. The effective pixels of the equipment are 24.20 megapixels. Image size is set to the aspect ratio of 3:2 which produces a ‘L’ type image with 6000 × 4000 pixels. The shutter speed of the equipment is 30-1/8000 s which is set to automatic during acquisition. All settings including autofocus points (AF) were set to automatic during the entire process.
Data source location	The geospatial coverage of the acquired data samples is from SRM Care Farm, Chengalpattu district, Tamil Nadu, India (603209). The latitude and longitude for collected data samples are 12.787003386255583 and 80.05900471261096. (Google Maps link: https://goo.gl/maps/Zq4yF8CddEyoZuWL6)
Data accessibility	Repository name: 1. SorghumWeedDataset_Classification 2. SorghumWeedDataset_Segmentation Data identification number: 1. DOI: 10.17632/4gkcyxjyss.1 2. DOI: 10.17632/y9bmtf4xmr.1 Direct URL to data: 1. https://data.mendeley.com/datasets/4gkcyxjyss/1 2. https://data.mendeley.com/datasets/y9bmtf4xmr/1

## 1 Value of the Data

• Knowing that data is the vital element of any deep learning/machine learning model, data samples in ‘SorghumWeedDataset_Classification’ and ‘SorghumWeedDataset_Segmentation’ are keenly acquired using a state-of-the-art instrument that records rich information about the research objects.
• Researchers, agriculturalists, and AI learners will benefit from these annotated datasets as they are made publicly available amidst the scarcity of datasets.
• These data are useful to train a deep learning/machine learning model that could differentiate a sorghum crop from its associated weeds. The trained model when integrated into the agricultural robot powers-up autonomous weeding.
• The two specified datasets can be used in model training, validating, and testing with or without pre-processing and augmentation.
• Novel methods to differentiate weeds from sorghum crops are appreciated as this could build a reliable and robust crop-weed detection model.

## 2 Data Description

This data article describes two datasets that have been created for weed identification from sorghum fields. ‘SorghumWeedDataset_Classification’ [1] is created to address the classification problems in which a data sample is either classified as a sorghum crop, grass, or a broadleaf weed. ‘SorghumWeedDataset_Segmentation’ [2] is created to address the localization, detection, and segmentation problems in which the research objects from a data sample are segmented pixel-wise. Classification dataset focuses on a primary research object whereas segmentation dataset focuses on multiple research objects.

### 2.1 SorghumWeedDataset_Classification

#### 2.1.1 Dataset creation

‘SorghumWeedDataset_Classification’ contains 1404 samples of sorghum samplings, 1467 samples of grasses, and 1441 samples of broadleaved weeds, making a sum of 4312 data samples. This dataset can be used for classification problems. Sorghum samplings are specified as class 0, grasses as class 1, and broadleaf weeds as class 2. All data samples are acquired during the initial stages of the crop and weed growth. The other stages of crop growth are not considered while creating this dataset to emphasize the early stages of crop growth.

#### 2.1.2 Data sample size

The average size of an original image is 13 MB with 6000 × 4000 pixels which made the overall size of the dataset size to be 41.7 GB. Deep learning models process the input images with a size of 224 × 224 pixels to reduce the computation complexity. Therefore, the original images are re-sized using image processing into the compactable size of 224 × 224 pixels without information loss. This allows the research community to reuse the dataset with no complexity. SorghumWeedDataset_Classification can be used as a standalone dataset or combined with other existing benchmark datasets.

#### 2.1.3 Splitting the dataset into TVT (Train-validation-test)

**Fig. 1:**
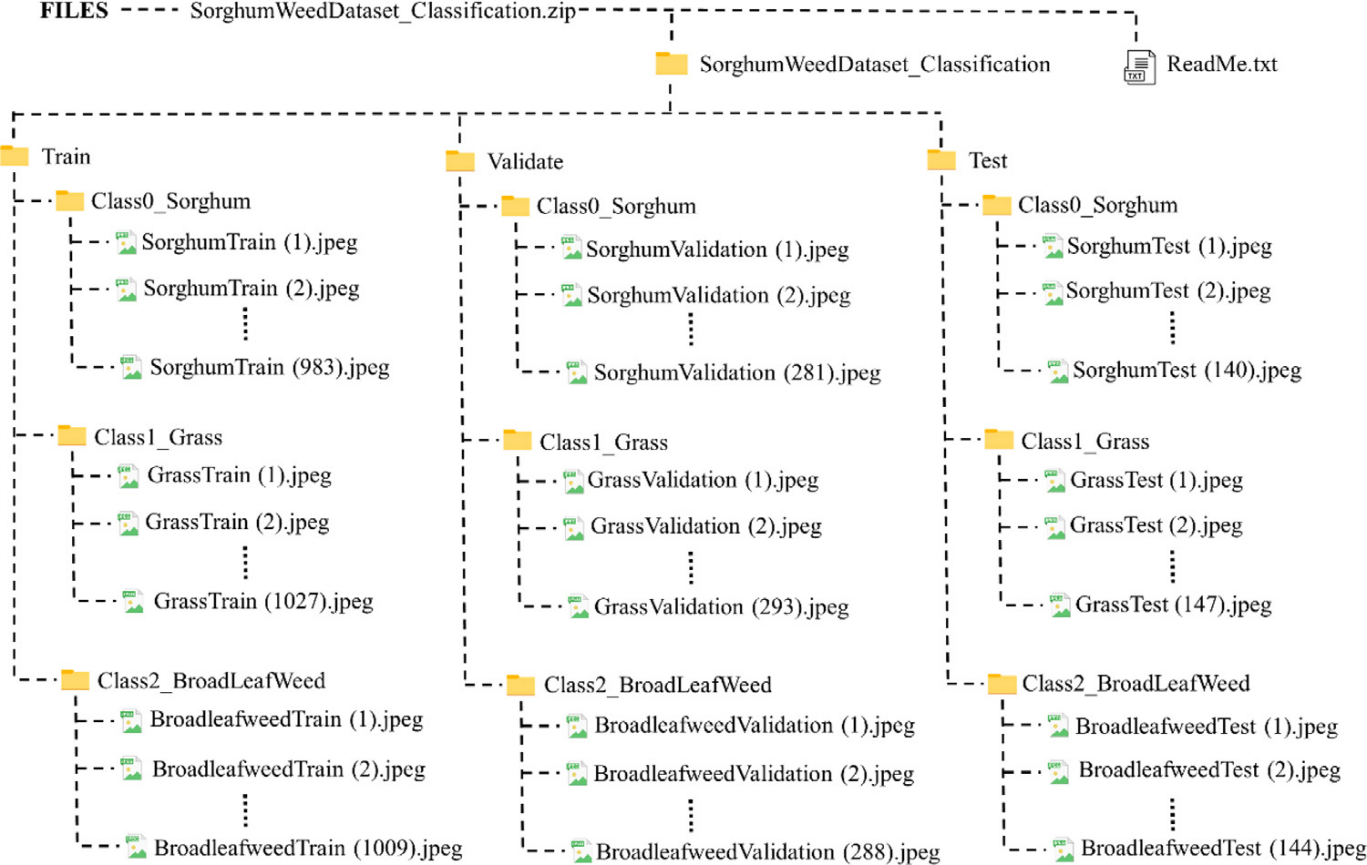
Directory structure of ‘SorghumWeedDataset_Classification’.

The TVT ratio considered for ‘SorghumWeedDataset_Classification’ is 7:2:1 and is detailed in Table 1. Fig. 1. describes the directory layout of the same whose root directory is ‘SorghumWeedDataset_Classification.zip’. It is a standard ZIP file found under the heading ‘Files’ in the link https://data.mendeley.com/datasets/4gkcyxjyss/1, which is downloaded when the download icon is clicked. The files are extracted to view the dataset directory ‘SorghumWeedDataset_Classification’ and ‘ReadMe.txt’. It does not need any third-party software to extract files, rather the operating system does it.

**Table 1:** ‘SorghumWeedDataset_Classification’ TVT split.

Class ID	Class name	No. of samples			
		Train (80 %)	Validate (20 %)	Test (10 %)	Total
Class 0	Sorghum	983	281	140	1404
Class 1	Grass	1027	293	147	1467
Class 2	BroadLeafWeed	1009	288	144	1441

Total		3019	862	431	4312

^a^All images are represented in JPEG.

### 2.2 Expected outcome

The deep learning/machine learning model built with ‘SorghumWeedDataset_Classification’ must be able to classify the correct class of a given input sample.

### 2.3 SorghumWeedDataset_Segmentation

#### 2.3.1 Dataset creation

‘SorghumWeedDataset_Segmentation’ contains 5555 manually pixel-wise annotated data segments from 252 data samples which contain sorghum, grass weed, and broadleaf weed samplings that can be used for object detection, instance segmentation, and semantic segmentation. The step-by-step- procedure to view the pixel-wise annotations of the data samples from ‘SorghumWeedDataset_Segmentation’ is shown in the video format and is included as a supplementary file with this article.

#### 2.3.2 Data sample and data segment size

The average size of an original image is 13 MB with 6000 × 4000 pixels which made the overall size of the dataset size to be 2.8 GB. Samples are not resized to retain the rich set of information which assisted in annotating data segments of plant length lesser than 0.5 cm.

#### 2.3.3 Splitting the dataset into TVT (Train-Validation-Test)

**Fig. 2:**
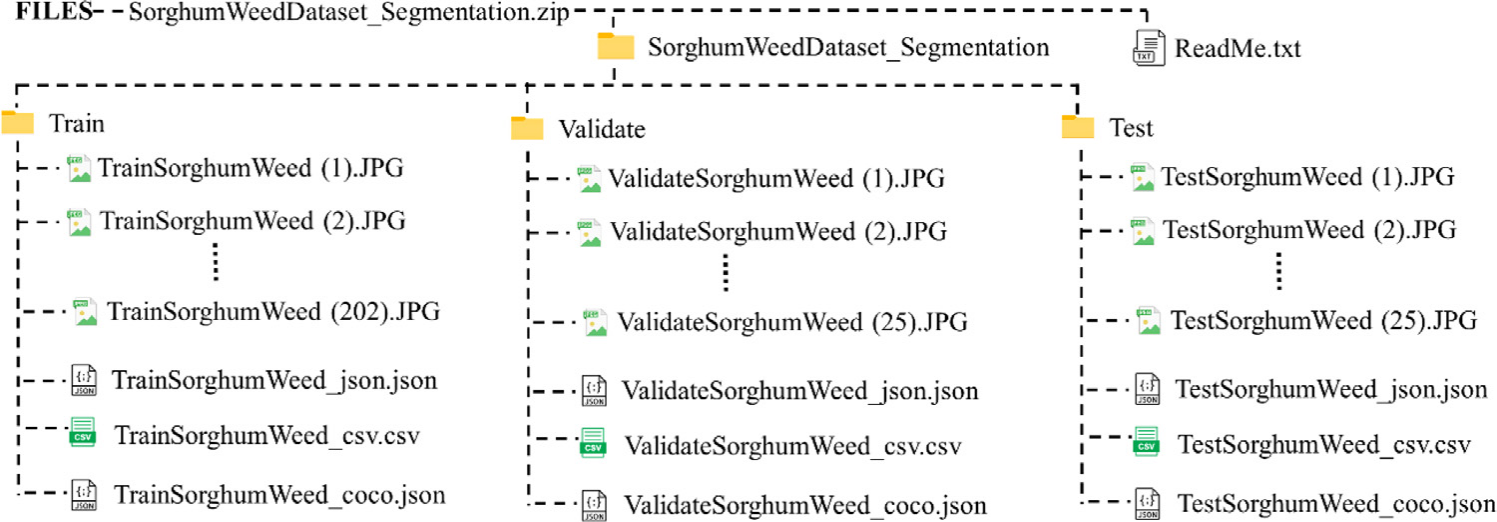
Directory structure of ‘SorghumWeedDataset_Segmentation’.

The TVT ratio considered for ‘SorghumWeedDataset_Segmentation’ is 8:1:1 and is detailed in Table 2. Table 3 tabulates the number of research objects in each category. All samples are manually annotated using VIA (VGG Image Annotator) software [3]. The respective annotation files for training, validation, and testing which are the ground truth of the segments are provided in JSON, CSV, and COCO format. Fig. 2. describes the directory layout of ‘SorghumWeedDataset_Segmentation’. The download method for this dataset is similar to the classification dataset, in which the ‘SorghumWeedDataset_Segmentation.zip’ ZIP file is downloaded and extracted to view the dataset directory.

**Table 2:** ‘SorghumWeedDataset_Segmentation’ image TVT split.

Train (80 %)	Validate (20 %)	Test (10 %)	Total
202	25	25	252

^a^All images are represented in JPG.

**Table 3:** ‘SorghumWeedDataset_Segmentation’ research objects TVT split.

Research objects / TVT	Sorghum	Grass	BLweed	Total
Train	1848	570	2544	4962
Validate	141	16	81	238
Test	172	23	160	355
Total	2161	609	2785	5555

#### 2.3.4 Expected outcome

The deep learning/machine learning model built with ‘SorghumWeedDataset_Segmentation’ will be able to localize and segment all the research objects present in a sample.

## 3 Experimental Design, Materials and Methods

### 3.1 Data acquisition requirements specification

#### 3.1.1 Specie selection

Sorghum, scientifically named ‘Sorghum bicolor L. Moench’ which belongs to the family ‘Poaceae’ is the specie selected as a primary research object. The objective of this research data acquisition is to address the weeding issues that occur in uniform crop spacing and random crop spacing fields. Sorghum seed sowing follows either broadcasting or dibbling methods of sowing. Sorghum is chosen as the primary research object as this easily grows in the southern part of India and requires minimum maintenance. Moreover, sorghum can yield high production just by adapting to several climates and temperatures with less input [4].

#### 3.1.2 Taxonomic coverage

**Fig. 3:**
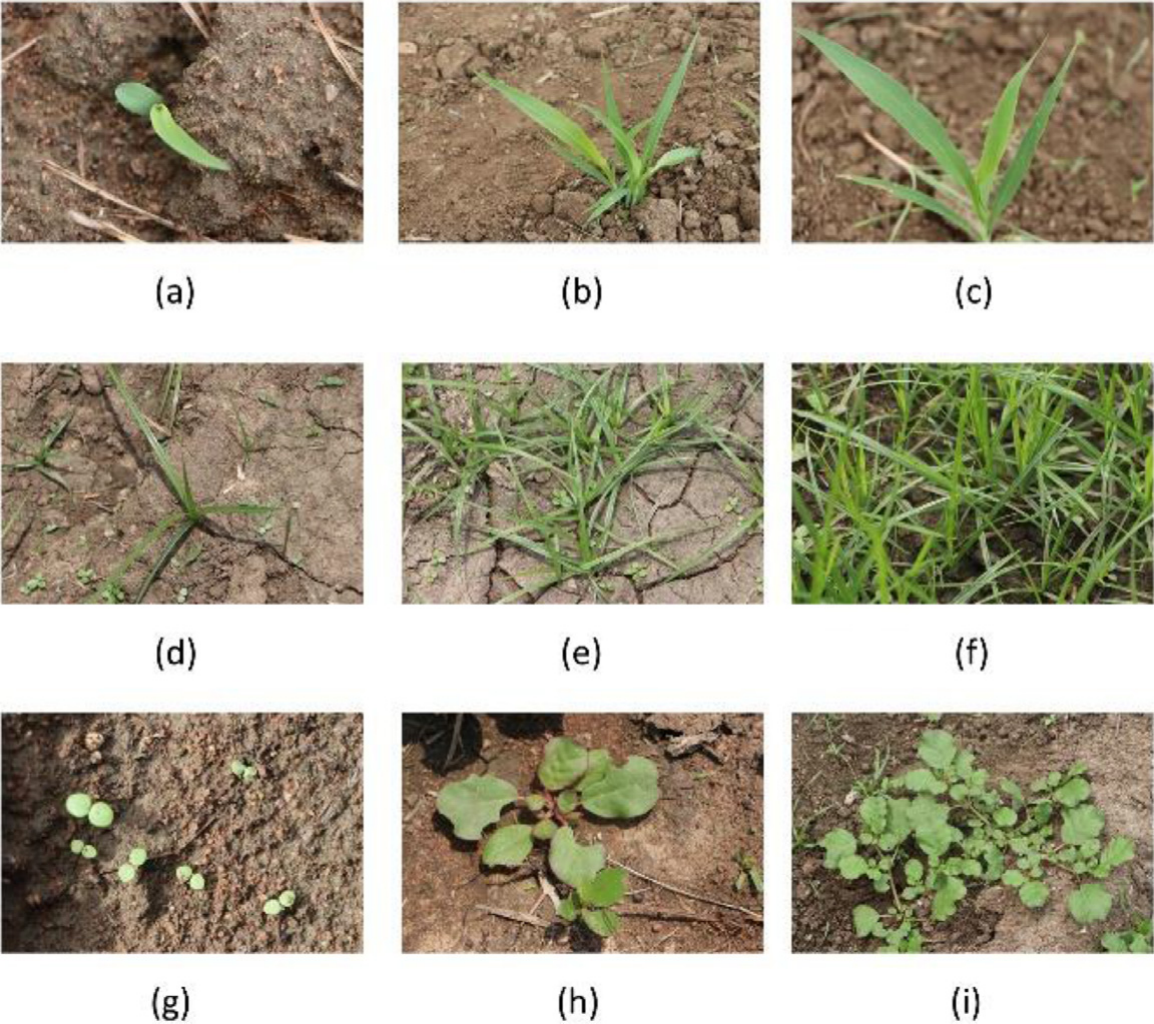
(a), (b), and (c) shows the various stages of Sorghum samplings. Grasses are shown in (d), (e), and (f). Broad leaf weeds are shown in (g), (h), and (i).

**Fig. 4:**
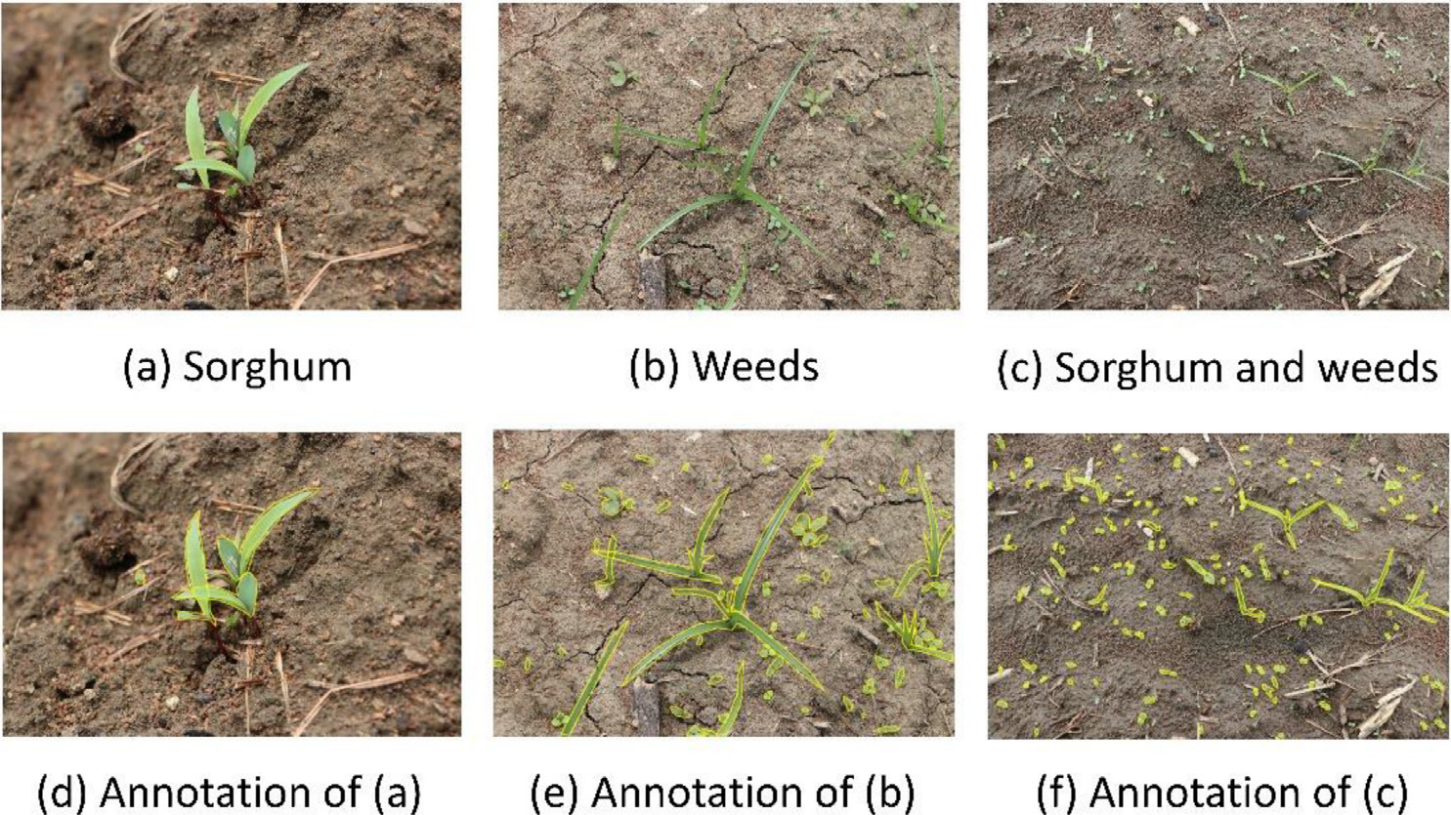
(a), (b), and, (b) present the data samples and (d), (e), and (f) show a representation of their respective annotations.

In the context of weed identification, any plant species other than the crop (sorghum in this data acquisition) is a weed [5]. Hence instead of focusing on every specific weed, a broad range of two weed categories are emphasized along with the crop. Sorghum, broad-leaved weeds (dicotyledonous), and grass weeds (monocotyledonous) are the three research objects focused on in this research data acquisition. We observed broadleaf weeds are dominantly present than grass weeds. Samples from ‘SorghumWeedDataset_Classification’ are exhibited in Fig. 3. Fig. 4 displays samples and their respective annotations from ‘SorghumWeedDataset_Segmentation’.

#### 3.1.3 Temporal coverage

The data samples are acquired during April and May 2023. To generalize the samples, data is acquired in three weather conditions - ‘Sunshine’, ‘High wind’, and ‘Gentle rain’ whose sample images are depicted in Table 4. We noticed some crops have been pushed down to the ground by ‘High wind’. On the day of data acquisition, the crop and weed samples are captured twice in different light conditions (morning light and afternoon light) which varied from day to day. In addition, each data sample is clicked from three different heights from 20 cm to 40 cm from the plant, and also by adjusting the camera lens accordingly. Ultimately, 5000+ samples of sorghum samplings, grasses, and broadleaf weeds are captured during the entire data acquisition process.

**Table 4:** Sample images acquired in three weather conditions.

## 4 Data Acquisition Platform

### 4.1 Initial land preparation and sowing methods

**Fig. 5:**
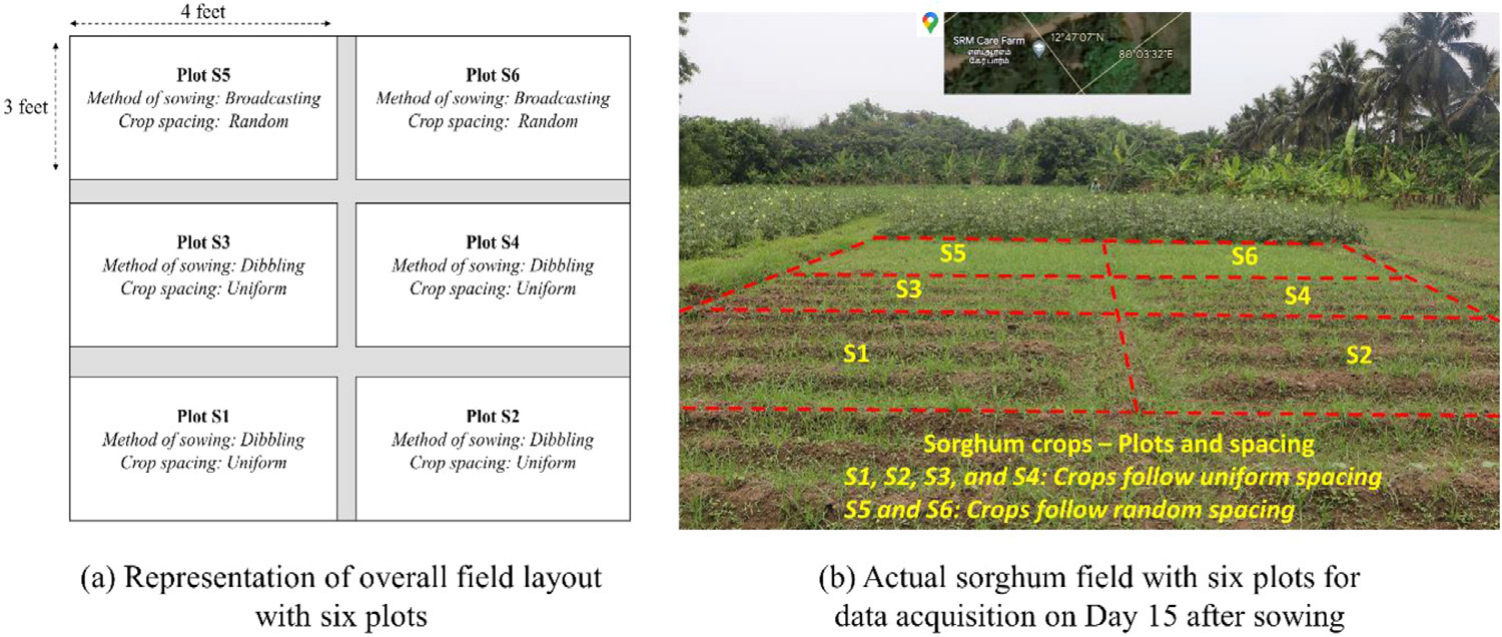
Plots S1 to S6 - Diagrammatic representation and actual field.

A total area of 871.2 square feet (2 cents) of agricultural field is set up for the establishment of sorghum crops to collect research data. The field is plowed on April 26th, 2023 followed by spreading manure onto farm fields. Seeds are sowed the next day, which is considered Day 1 in this research. The field is divided into six plot arrangements named S1, S2, S3, S4, S5, and S6 respectively. Each plot is four feet in length and three feet in breadth. Plots S1, S2, S3, and S4 follow the dibbling method of sowing, and plots S5 and S6 follow the broadcasting method of sowing seeds. Fig. 5 shows the overall diagrammatic representation of the field layout and the actual field plots used for data acquisition.

### 4.2 Planting pattern for S1, S2, S3, and S4 with uniform spacing

**Fig. 6:**
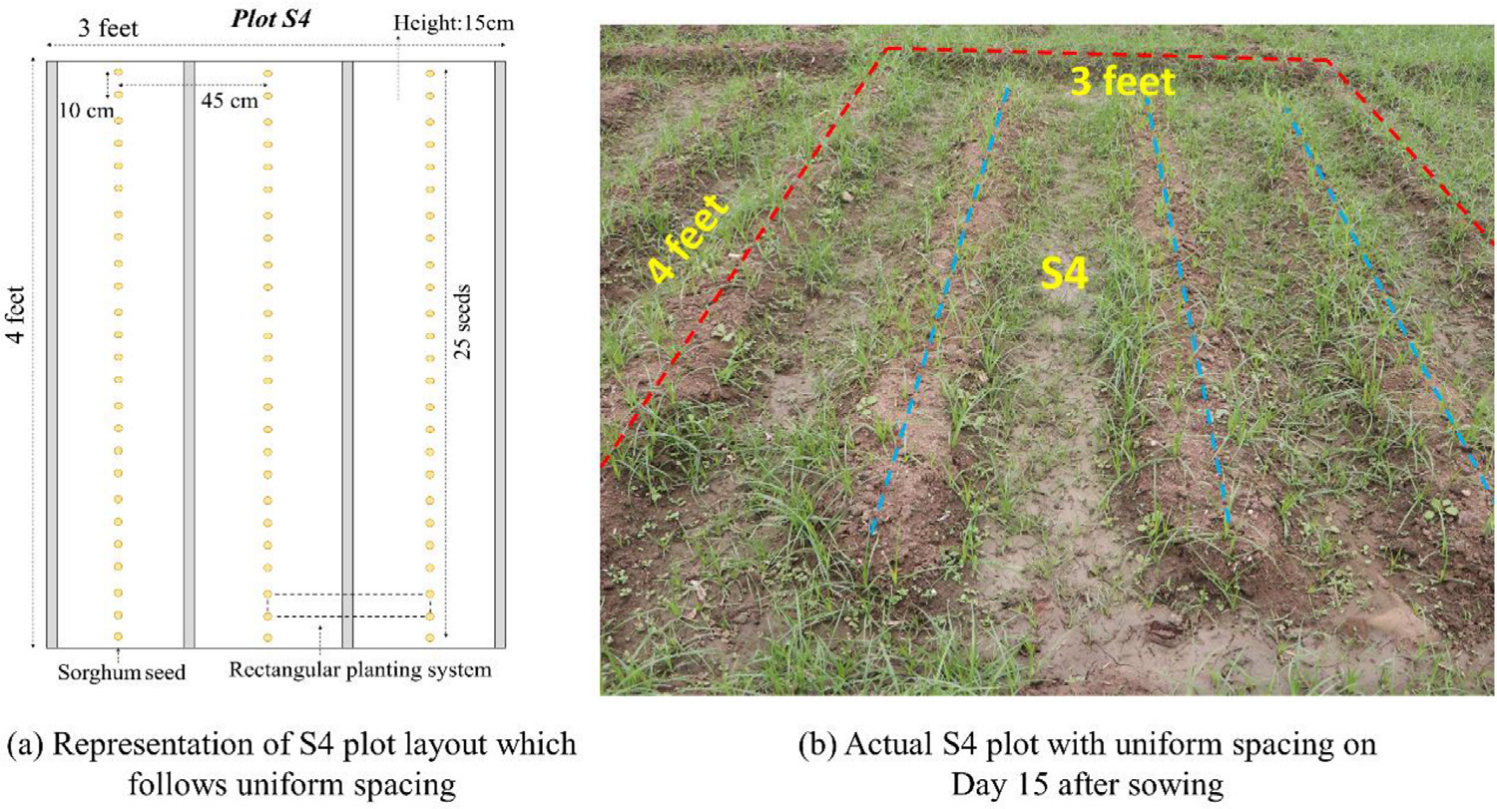
S4 plot layout - Diagrammatic representation and actual field.

The distribution of sorghum seeds in S1, S2, S3, and S4 is uniform which resulted in regular spacing intervals between the sorghum crops. There are three rows in each plot, with each row raised to a height of 15 cm. Each row has 25 sorghum seeds dibbled into it at a depth of 5 cm, following the rectangular planting system. The inter-row spacing and inter-crop spacing of the seeds are 45 cm and 10 cm respectively. 12 rows from S1, S2, S3, and S4 have produced an overall sorghum population of 300. Fig. 6 shows the representation layout of plot S4 and the actual S4 field plot which follows uniform crop spacing.

### 4.3 Planting pattern for S5 and S6 with random spacing

The distribution of sorghum seeds in S5 and S6 is not uniform as they are scattered randomly. This resulted in a non-uniform spacing between sorghum crops, moreover, crop clusters are also observed. S5 and S6 produced a sorghum population between 80 and 100.

## 5 Data Acquisition Methodology

### 5.1 Data acquisition with growth stages of sorghum

**Fig. 7:**
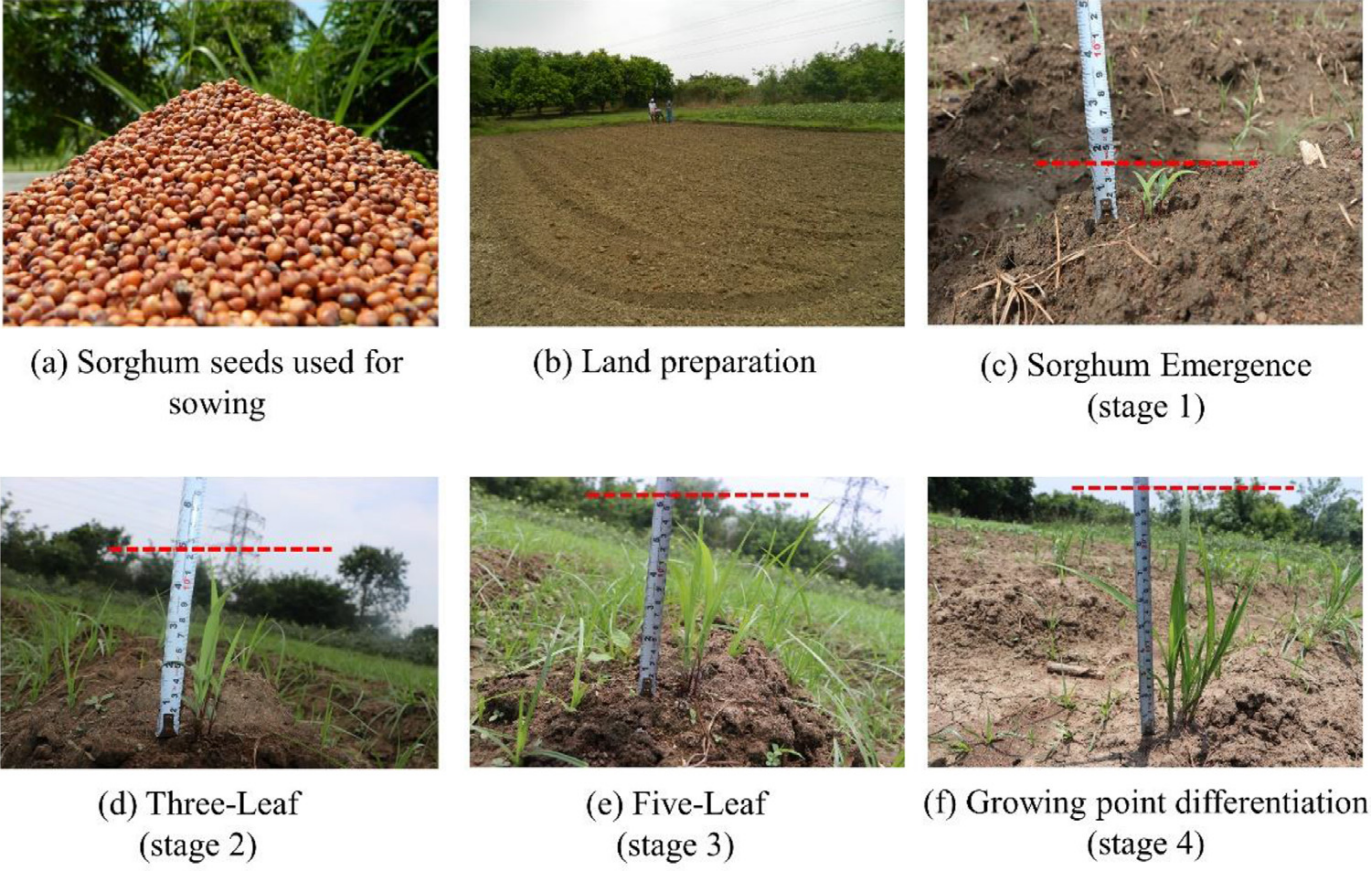
Various growth stages of the sorghum crop during data acquisition.

**Fig. 8:**
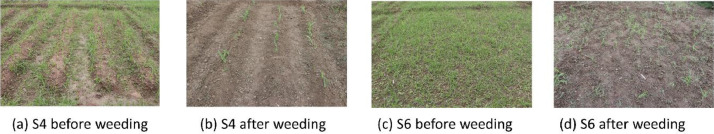
(a) and (b) displays uniform spacing plot before and after weeding, (c) and (d) displays random spacing plot before and after weeding.

Sorghum has 10 stages of crop growth with a maximum duration of 120 days. After sowing sorghum seeds, the first 45 days are known to be the critical period of weed competition [6]. Hence, the data acquisition has focused only on the first four stages of crop growth when there is high competition between sorghum and its weeds. Emergence (stage 1) was observed on Day 6 after sowing, Three-Leaf (stage 2) was observed on Day 9 followed by Five-Leaf (stage 3) on Day 15, and Growing point differentiation(stage 4) on Day 23. The first hand weeding is done on Day 17 followed by second hand weeding on Day 40, to avoid occlusion of crops and weeds which interrupts data acquisition. Data acquisition is carried out even after weeding. Fig. 7 illustrates the various stages during the acquisition process and Fig. 8 displays the plots S4 and S6 before and after the first weeding.

## Limitations

**Fig. 9:**
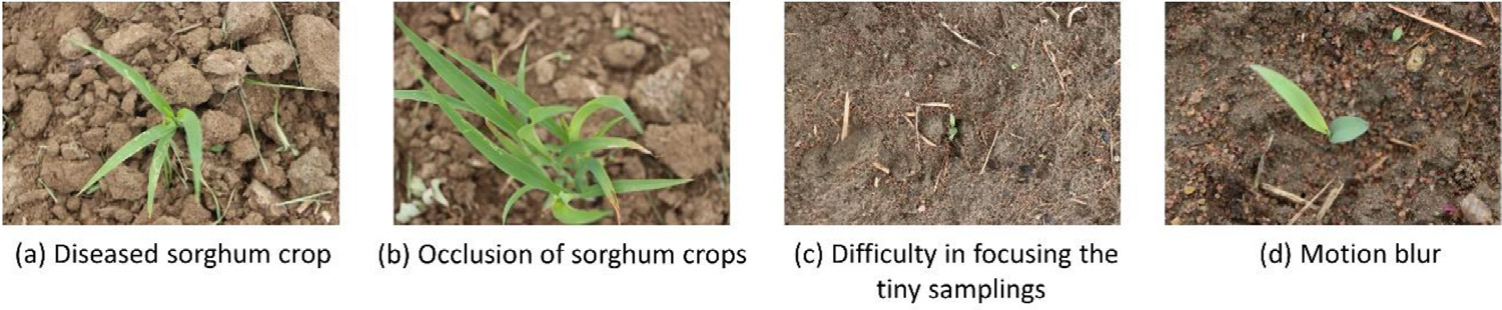
Challenges faced during the data acquisition process.

Data acquisition from agricultural fields for computer vision applications is a challenging task due to several factors. The first concern is the collection of a huge number of data samples for creating a large dataset to train the model. Another challenge is acquiring data during the early stages of crop growth since it is harder to focus on the research objects. Acquisition with various climatic conditions, object occlusion, shadowing due to different lighting conditions, motion blur, and diseased leaves are other accompanying complexities we faced during the acquisition process. Data labeling, segregation into different classes, and pixel-wise object annotation is a time-consuming task. Fig. 9 illustrates the challenges faced during the data acquisition process.

## Ethics Statement

The authors have read and followed the ethical requirements for publication in Data in Brief and confirmed that the current work does not involve human subjects, animal experiments, or any data collected from social media platforms.

## CRediT authorship contribution statement

**Michael J. Justina:** Methodology, Resources, Data curation, Writing – original draft, Visualization, Funding acquisition. **M. Thenmozhi:** Conceptualization, Validation, Formal analysis, Investigation, Writing – review & editing, Supervision, Project administration.

## Data Availability

SorghumWeedDataset_Classification (Original data) (Mendeley Data) SorghumWeedDataset_Segmentation (Original data) (Mendeley Data)

## Appendix Supplementary materials

Image, video 1
